# Phytochemical, Antimalarial, and Acute Oral Toxicity Properties of Selected Crude Extracts of Prabchompoothaweep Remedy in *Plasmodium berghei*-Infected Mice

**DOI:** 10.3390/tropicalmed7120395

**Published:** 2022-11-23

**Authors:** Walaiporn Plirat, Prapaporn Chaniad, Arisara Phuwajaroanpong, Abdi Wira Septama, Chuchard Punsawad

**Affiliations:** 1 Department of Medical Sciences, School of Medicine, Walailak University, Nakhon Si Thammarat 80160, Thailand; 2 Research Center in Tropical Pathobiology, Walailak University, Nakhon Si Thammarat 80160, Thailand; 3 Research Center for Pharmaceutical Ingredient and Traditional Medicine, National Research and Innovation Agency (BRIN), Cibinong Science Center, Bogor 16915, Indonesia

**Keywords:** antimalarial activity, toxicity, Prabchompoothaweep remedy, *Myritica fragrans*, *Atractylodes lancea*, malaria

## Abstract

Malaria remains a life-threatening health problem and encounters with the increasing of antimalarial drug resistance. Medicinal plants play a critical role in synthesizing novel and potent antimalarial agents. This study aimed to investigate the phytochemical constituents, antiplasmodial activity, and evaluate the toxicity of crude ethanolic extracts of *Myristica fragrans*, *Atractylodes lancea*, and Prabchompoothaweep remedy in a mouse model. The phytochemical constituents were characterized by liquid chromatography-mass spectrometry (LC-MS). Antimalarial efficacy against *Plasmodium berghei* was assessed using 4-day suppressive tests at doses of 200, 400, and 600 mg/kg body weight. Acute toxicity was assessed at a dose of 2000 mg/kg body weight of crude extracts. The 4-day suppression test showed that all crude extracts significantly suppressed parasitemia (*p* < 0.05) compared to the control group. Higher parasitemia suppression was observed both in Prabchompoothaweep remedy at a dose of 600 mg/kg (60.1%), and *A. lancea* at a dose of 400 mg/kg (60.1%). The acute oral toxicity test indicated that the LD_50_ values of all extracts were greater than 2000 mg/kg and that these extracts were not toxic in the mouse model. LC-MS analysis revealed several compounds in *M. fragrans*, *A. lancea*, and Prabchompoothaweep remedy. For quantitative analysis, 1,2,6,8-tetrahydroxy-3-methylanthraquinone 2-*O*-b-D-glucoside, chlorogenic acid, and 3-O-(beta-D-glucopyranosyl-(1->6)-beta-D-glucopyranosyl) ethyl 3-hydroxyoctanoate were found in *A. lancea*, while (7′x,8′x)-4,7′-epoxy-3,8′-bilign-7-ene-3,5′-dimethoxy-4′,9,9′-triol, edulisin III, and tetra-hydrosappanone A trimethyl ether are found in *M. fragrans*. 6′-O-Formylmarmin was present in the Prabchompoothaweep remedy, followed by pterostilbene glycinate and amlaic acid. This study showed that the ethanolic extracts of *A. lancea* and Prabchompoothaweep remedy possess antimalarial activity against *Plasmodium berghei*. None of the extracts had toxic effects on liver and kidney function. Therefore, the ethanolic extract of *A. lancea* rhizome and Prabchompoothaweep remedy could be used as an alternative source of new antimalarial agents. Further studies are needed to determine the active compounds in both extracts.

## 1. Introduction

Malaria is one of the most serious and life-threatening infectious diseases caused by protozoan parasites of the *Plasmodium* genus. It is responsible for the high rates of mortality and morbidity in the tropical and subtropical regions of the world, where the climate is suitable for parasite development [1]. According to the World Health Organization report in 2021, there were approximately 241 million cases of malaria which caused 0.6 million deaths worldwide [2]. The mortality rate from malaria has been reduced in recent years due to extensive malaria control through the use of insecticide-impregnated bed nets and treatment with artemisinin derivatives; however, the state of artemisinin resistance, the standard drug for treating malaria, is of great concern [3]. Artemisinin-based combination therapies (ACTs) are the first-line drugs for malaria in a large majority of endemic countries, and intravenous artesunate is usually used for the treatment of severe malaria [4]. Although ACTs act as a fast-acting artemisinin derivative and a slow-acting combined drug, the efficacy of ACTs is limited by long-lasting parasite clearance, contributing to ACT failure [5,6]. Furthermore, *Plasmodium falciparum* infection has become resistant to almost all available antimalarial drugs, which is estimated to be 10% in Southeast Asia and 93% in Thailand [7]. To manage this pathology, a new antimalarial compound that is safer, more effective than older drugs, and has a novel mode of action is urgently required.

For centuries, plants and herbs have been an important source of drugs being developed to provide a potential treatment for many diseases. Plants contain a large number of bioactive molecules and are a valuable source of pharmacotherapeutics [8,9]. Furthermore, antimalarial drugs, especially quinine and artemisinin, are derived from traditional medicines and plant extracts [10]. Therefore, natural plants are a good source of inspiration in searching for a new antimalarial agent.

Prabchompoothaweep is a traditional Thai medicine that is part of the National List of Essential Medicines (NLEM), which includes 23 herbs [11]. The bioactivity of the ethanolic extract of Prabchompoothaweep remedy, including antiallergic activity, anti-inflammation, and antioxidant activities, has been reported. According to the NLEM, the Prabchompoothaweep remedy is usually suggested to be useful for the treatment of many types of fever, including malaria-like symptoms such as intermittent fever and common cold [12]. In addition, Prabchompoothaweep remedy and two-component plants of Prabchompoothaweep remedy, *Myristica fragrans*, and *Atractylodes lancea*, have been reported to show in vitro antimalarial activity. From our previous studies, the in vitro antimalarial activity of the ethanolic extracts of the Prabchompoothaweep remedy, *M. fragrans*, and *A. lancea*, displayed antimalarial activity (IC_50_ = 14.13 µg/mL, 5.96 µg/mL, and 7.73 µg/mL, respectively) (unpublished data). *M. fragrans* is an aromatic evergreen tropical tree belonging to the Myristicaceae family [13]. *M. fragrans* has been used to treat several diseases. In particular, the mace part, which is an aril of *M. fragrans*, has been used for asthma, fever, and gastrointestinal treatment in Ayurvedic medicine [14]. Furthermore, *M. fragrans* has been suggested to have various medicinal properties, such as antimicrobial, chemoprotective, antioxidant, anti-inflammatory effects, anti-atherosclerosis, and behavioral effects [15]. *A. lancea* belongs to the Asteraceae (Compositae) family [16]. *A. lancea* has been used to treat rheumatic diseases, digestive disorders, night blindness, and influenza [17]. The pharmacological properties of rhizomes, including anti-cancer, anti-inflammatory, and antimicrobial activities and activities on the central nervous, cardiovascular, and gastrointestinal systems, have been investigated [18]. Prabchompoothaweep remedy and two-component plants have shown good in vitro antimalarial activity. Therefore, the Prabchompoothweep remedy and its two components are good candidates for further investigation of the in vivo antimalarial activity.

Based on ethnobotanical evidence and our in vitro study of antimalarial activity and toxicity, ethanolic mace extracts of *M. fragrans*, ethanolic rhizome extract of *A. lancea*, and ethanolic crude extract of Prabchompoothaweep remedy were found to have good activity against parasite infection without cytotoxicity to Vero cells. Therefore, this study aimed to investigate the potential antimalarial activity and toxicological assessment of two crude extracts from the Prabchompoothaweep remedy in a mouse model. Furthermore, the phytochemical content of selected crude extracts of the Prabchompoothaweep remedy was explored to understand the origin of the bioactivity.

## 2. Materials and Methods

### 2.1. Plant Collection

The dried arils (mace) of *M. fragrans*, dried rhizome of *A. lancea*, and Prabchompoothaweep remedy were purchased from a traditional Thai drug store in the Nakhon Si Thammarat region of Thailand. The authorization for plant materials complied with the relevant guidelines and regulations of the Plant Varieties Protection, Department of Agriculture, Ministry of Agriculture and Cooperatives, Thailand. The botanical identification of the plant samples was confirmed by a botanist at the School of Pharmacy, Walailak University. Specimens with voucher numbers for *M. fragrans* (SMD177004003-2) and *A. lancea* (SMD072010001) were deposited in the School of Medicine, Walailak University.

### 2.2. Preparation of Plant Extracts

First, the plant samples were powdered using a herb grinder (Jincheng, Model; SF, China). *M. fragrans* aril powder (60 g), *A. lancea* rhizome powder (60 g), and Prabchompoothaweep remedy powder (60 g) were soaked in 600 mL of 95% ethanol for 72 h at room temperature (1:10 (*w*/*v*) ratio). The mixed solutions were filtered using gauze and Whatman filter No. 1. The unfiltered residues were remacerated in 95% ethanol for 72 h. This procedure was repeated two times. The filtered solutions were combined and concentrated using a rotary evaporator (Buchi^®^ rotary evaporator, Model R-210, Shanghai, China). The residues were then dried in a water bath at 60 °C. Finally, the dried crude extracts of *M. fragrans* aril, *A. lancea* rhizome, and Prabchompoothaweep remedy were stored in a refrigerator at 4 °C until use. For animal experiments, each crude extract was dissolved in 7% Tween 80 and 3% ethanol in distilled water to obtain the working concentration.

### 2.3. Phytochemical Screening

The ethanolic extract was qualitatively investigated to reveal the presence of phytochemical constituents, including flavonoids, terpenoids, alkaloids, tannins, anthraquinones, cardiac glycosides, saponins, and coumarins. These were identified by characteristic color changes using standard procedures [19,20,21].

### 2.4. Liquid Chromatography-Quadrupole Time-of-Flight Mass Spectrometry (LC-QTOF MS) Analysis

The metabolite profiles of *M. fragrans* extract, *A. lancea* extract, and Prabchompoothaweep remedy extract were determined using by an ultra-high performance liquid chromatography (UHPLC) instrument equipped with an electrospray ionization source (ESI). The UHPLC system consisted of a Zorbax Eclipse Plus C18 Rapid Resolution HD column (150 mm length × 2.1 mm inner-diameter, particle size 1.8 µm) with an LC-QTOF MS instrument (1290 Infinity II LC-6545 Quadrupole-TOF, Agilent Technologies, Santa Clara, CA, USA). The mobile phase comprised solvent A (0.1% formic acid in water) and solvent B (acetonitrile). The volume of injection was 2.0 µL, and the column temperature was set at 25 °C. Qualitative analysis of LC-MS/MS was performed in negative ion mode with a scanning range from m/z 100 to 1200 using a Dual AJS ESI ion source. The phytochemical compounds in the extract samples were identified by comparing the retention time, mass data, and fragmentation patterns with known compounds in the library search of the Mass Hunter METLIN database (Agilent Technologies). The compound selection was selected and identified from the peak with 90% similarity in the database.

### 2.5. Animals and Rodent Parasites

Healthy male Institute of Cancer Research (ICR) mice aged 6–8 weeks, weighing 20–30 g, were purchased from Nomura Siam International Co., Ltd., Bangkok, Thailand. The animals were housed and acclimatized for 7 days under standard and constant laboratory conditions (22 ± 3 °C, 50–60% humidity and 12 h light/dark cycles) with free access to food and clean water. The animal care staff controlled the hygiene by cleaning and removing waste from the cages daily. The mice were handled according to the international guidelines for the animals used in the experiments. The wild-type rodent *Plasmodium berghei* ANKA strain was obtained from Biodefense and Emerging Infections Research Resources Repository (BEI Resources), National Institute of Allergy and Infectious Diseases (NIAID), and National Institute of Health (NIH), which was received from Thomas F. McCutchan. Mouse donors were injected with *P. berghei*-infected red blood cells via an intraperitoneal route. When the mouse donors had parasitemia levels of 20–30%, blood was drawn from the heart by cardiac puncture and kept in a heparinized tube for injection into experimental mice.

### 2.6. Animal Grouping and Dosing

For Peter’s 4-day suppressive test, infected male ICR mice were randomly divided into 12 groups of five mice per group. Group 1 (infected control mice) was administered a mixture of 7% Tween 80 and 3% ethanol in distilled water. Groups 2 and 3 (positive control) received 6 mg/kg body weight of artesunate (Art) and 25 mg/kg body weight of chloroquine (CQ), respectively. Groups 4, 5, and 6 were administered 200, 400, and 600 mg/kg body weight of *M. fragrans* crude extract, respectively. Groups 7, 8, and 9 were administered 200, 400, and 600 mg/kg body weight *A. lancea* crude extract, respectively. Groups 10, 11, and 12 were administered 200, 400, and 600 mg/kg body weight of Prabchompoothaweep remedy crude extract, respectively. Dosage selection was chosen based on the results of oral acute toxicity and preliminary results were obtained for the extracts. For oral acute toxicity testing, mice were randomly assigned to five groups of five mice each. Group 1 (untreated control group) received no treatment; Group 2 (negative control group) was treated with a mixture of 7% Tween 80 and 3% ethanol in distilled water; Group 3 was treated with a dose of 2000 mg/kg body weight of *M. fragrans* crude extract; Group 4 was treated with a dose of 2000 mg/kg body weight of *A. lancea* crude extract; and Group 5 was treated with a dose of 2000 mg/kg body weight of Prabchompoothaweep remedy crude extract. Acute toxicity in mice was induced by oral administration.

### 2.7. Four-Day Suppressive Test (Peter’s Test)

The protocol for a 4-day suppressive test was evaluated according to previous studies [22]. First, all mice were injected with 0.2 mL of 1 × 10^7^ infected blood cells (intraperitoneally); 3 h after infection, the mice in each group were treated with the crude extract as described above and continued to be treated for 3 consecutive days (24, 48, and 72 h after infection). Treatment was administered via oral gavage to mimic the traditional route of administration. On day 5 post-infection, blood was collected from the vascular tail vein to prepare a thin blood smear film. Thin blood smears were stained with 10% Giemsa solution (Biotech Reagent Company Limited, Bangkok, Thailand) to evaluate parasitemia. Parasitemia was observed under a light microscope (Olympus, model: CX-31, Tokyo, Japan) with a 100X objective lens. The percentage of parasitemia was determined from five different fields with an estimated 300 red blood cells per field, and the percentage of parasitemia was calculated using the following formula:

$$\%\mathrm{parasitemia}=\frac{\mathrm{number}\;\mathrm{of}\;\mathrm{parasitized}\;\mathrm{red}\;\mathrm{blood}\;\mathrm{cells}}{\mathrm{number}\;\mathrm{of}\;\mathrm{total}\;\mathrm{red}\;\mathrm{blood}\;\mathrm{cells}}$$

The percentage of parasitemia suppression was calculated using the following formula:

$$\%\mathrm{suppression}=\frac{\left[A-B\right]}{A}\;\times \;100$$

where A is the mean percentage of parasitemia in the infected control group and B is the mean percentage of parasitemia in each treatment group.

### 2.8. Pack Cell Volume (PCV)

The effectiveness of the crude extracts in preventing hemolysis due to increasing parasite levels was measured using PCV. The tail vein of mice was cut to collect the blood, and the blood was kept in heparinized micro-hematocrit capillary tubes by filling them up to 3/4. One side of the capillary was plugged with clay. The capillary tubes were then centrifuged at 9520× *g* for 5 min with the sealed ends outwards. The PCV of each mouse was determined on day 0 before infection with *P. berghei* and day 4 after treatment.

### 2.9. Acute Toxicity Measurement

The oral acute toxicity of ethanolic crude extracts of *M. fragrans*, *A. lancea*, and Prabchompoothaweep remedy was investigated in male ICR mice according to the standard guidelines of the Organization for Economic Co-operation and Development (OECD) [23]. Twenty-five mice were randomly separated into five groups of five, which were explained in the animal grouping and dosing section. On day 1, the mice were not allowed to obtain food and water for 3 h before treatment. Subsequently, the mice in the treatment group were orally administered a single dose of 2000 mg/kg body weight of *M. fragrans*, *A. lancea*, or Prabchompoothaweep remedy extract. A mixture of 7% Tween 80 and 3% ethanol in distilled water served as the negative control, and untreated mice served as the control. Three hours after treatment, the mice were noted to have physical and behavioral changes such as muscle tone, mood, sleep, excretion, appetite, and hair erection. The animals were observed daily for 14 days. Food and water intake were recorded daily. The body weight of the mice was measured on days 0 and 14 using a sensitive digital weighing balance (Mettler Toledo, model: ML3002E, Bekasi City, Indonesia). On day 14, the mice were anesthetized with 50 mg/kg body weight sodium pentobarbital (Ceva Sante Animale, Maassluis, The Netherlands) by intraperitoneal injection. After anesthetization, mouse blood was collected for biochemical analysis. Liver and kidney tissues were harvested for histopathological examination using hematoxylin and eosin (H&E) staining.

### 2.10. Biochemical Analysis

Blood samples from the acute toxicity test group were collected from the heart using a cardiac puncture technique. Blood was centrifuged at 3000× *g* for 5 min to separate the plasma, which was collected to evaluate liver and kidney function. Liver and kidney functions were tested for biochemical parameters, including aspartate aminotransferase (AST), alanine aminotransferase (ALT), alkaline phosphate (ALP), blood urea nitrogen (BUN), and creatinine levels, using an AU 480 chemistry analyzer (Beckman Coulter, Brea, CA, USA).

### 2.11. Histopathological Examination

Histopathological investigation was performed using a standard laboratory procedure, as previously reported [24,25,26]. The tissues were fixed in 10% (*v*/*v*) formalin at room temperature, dehydrated with a series of alcohol concentrations, cleared with xylene, and embedded in paraffin. After tissue processing, the liver and kidney tissues were cut to 5 µm thickness using a microtome, stained with hematoxylin and eosin solution, and evaluated under a light microscope by two independent observers blinded to the condition groups.

### 2.12. Statistical Analysis

Statistical analysis was performed with SPSS statistical software version 23 (IBM, Armonk, NY, USA). Quantitative data were presented as means ± standard errors of the means (means ± SEMs). The Kolmogorov–Smirnov test was used to assess the normal distribution of each parameter. Differences in the mean parameters between the groups, such as the percentage of parasitemia, percentage of suppression, food and water consumption, body weight, and liver and lung biochemical parameters, were analyzed with a one-way analysis of variance followed by a post-hoc Tukey’s multiple comparison test. A *p*-value of less than 0.05 was considered statistically significant for all tests.

## 3. Results

### 3.1. Percentage Yield and Phytochemical Screening of Ethanolic Crude Extracts

The percentage yield of the ethanolic mace extract of *M. fragrans*, rhizome extract of *A. lancea*, and Prabchompoothaweep remedy was 20.71%, 22.11%, and 5.43%, respectively. The phytochemical constituents included flavonoids, terpenoids, alkaloids, tannins, and coumarins (Table 1).

### 3.2. LC-QTOF-MS Analysis

**Figure 1:**
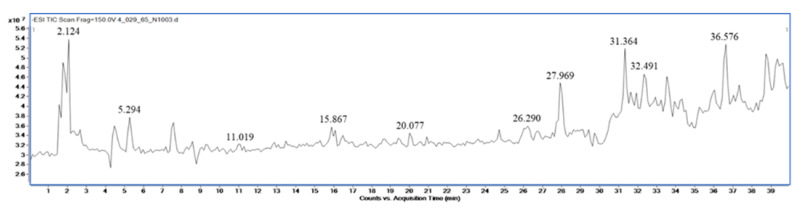
LC-MS chromatogram of ethanolic *M. fragrans* mace extract.

**Figure 2:**
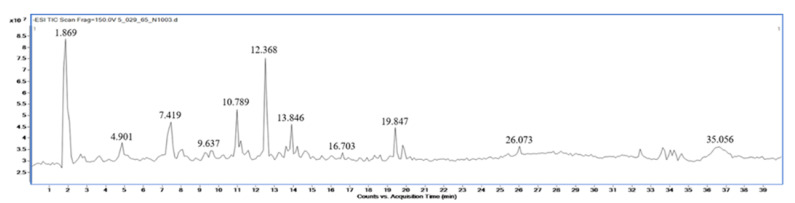
LC-MS chromatogram of ethanolic *A. lancea* rhizome extract.

**Figure 3:**
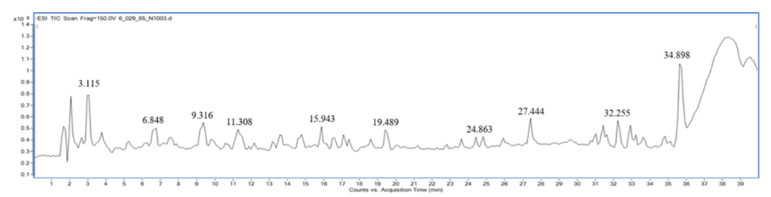
LC-MS chromatogram of ethanolic extract of Prabchompoothaweep remedy.

Qualitative analysis of the compounds in extracts of *M. fragrans*, *A. lancea*, and Prabchompoothaweep remedy was performed using LC-QTOF-MS in negative mode. Metabolite profiling of the crude extract compounds was performed using a database of well-known compounds in the Library METLIN database. The complete list of compounds detected using LC-QTOF-MS is given in Table 2, Table 3 and Table 4 and supported by Figure 1, Figure 2 and Figure 3.

### 3.3. Four-Day Suppressive Test

The antimalarial activity of *M. fragrans* extract, *A. lancea* extract, and Prabchompoothaweep remedy extract against *P. berghei* ANKA was measured using a 4-day suppressive test. Animals in each condition were treated with daily doses of crude extracts at 200, 400, and 600 mg/kg body weight by an oral route. The results showed that mice treated with extracts of *M. fragrans* and Prabchompoothaweep remedy showed significant suppression of parasitemia in a dose-dependent response (*M. fragrans*: 38.32, 44.17, and 46.86, respectively; and Prabchompoothaweep remedy: 39.18, 48.35, and 60.11, respectively) compared to the negative control group (*p* < 0.05). The *A. lancea* group also showed suppressed parasites compared to the negative control group (*p* < 0.05), especially at a dose of 400 mg/kg body weight. Parasite levels decreased after treatment with the ethanolic extract of *M. fragrans*, *A. lancea*, and Prabchompoothaweep remedy. However, all treatment groups in the crude extract did not completely suppress parasitemia, whereas the parasites were suppressed by more than 95% in the positive control groups (6 mg/kg body weight artesunate and 25 mg/kg body weight chloroquine). Parasite levels and parasite suppression are shown in Table 5.

### 3.4. PCV

The effects of ethanolic extracts of *M. fragrans*, *A. lancea*, and Prabchompoothaweep remedy on PCVs are presented in Table 6. In the positive treatment control group (artesunate and chloroquine), there was a significant decrease in PCV compared with the negative control group (*p* > 0.05). The PCV loss was protected by 200, 400, and 600 mg/kg doses of crude extracts compared to the negative control group. However, the protection of crude extracts did not significantly reduce PCV loss at any dose of crude extract compared to the negative control group (*p* < 0.05).

### 3.5. Acute Oral Toxicity Test

#### 3.5.1. Physical Activity and Behavior, Food and Water Uptake, and Body Weight

On the first day of the experiment, mice were administered a single dose of 2000 mg/kg *M. fragrans* ethanolic extract, *A. lancea* ethanolic extract, or Prabchompoothaweep remedy ethanolic extract. Physical activity and behavioral changes were observed for 14 consecutive days after treatment. The results showed no signs or symptoms of toxicity, such as rigidity, mood changes, ataxia, abnormal sleep, diarrhea, vomiting, consumption changes, and hair erection, during the experiment period. The mice in the acute toxicity test did not show mortality within the first 24 h or 14 days of treatment. Therefore, lethal doses of *M. fragrans* extracts, *A. lancea* extracts, or Prabchompoothaweep remedy extracts are greater than 2000 mg/kg body weight. According to water and food consumption in acute toxicity tests after treatment with ethanolic extracts, the mean water and food consumption of mice in the treatment groups treated with a single dose of 2000 mg/kg body weight of *M. fragrans* extract, *A. lancea* extract, Prabchompoothaweep remedy extract, and those in the 7% Tween 80 group (negative control group) did not show significant differences compared to those of mice in the control group (untreated group) (*p* > 0.05) (Table 7). Furthermore, the body weight changes in mice treated with 2000 mg/kg crude extracts and 7% Tween 80 were not significantly different from those in the control group (*p* > 0.05) (Table 8) at week 2 after receiving crude extracts.

#### 3.5.2. Biochemical Assessment of Liver and Kidney Functions

The levels of liver function, such as AST, ALT, and ALP, in mice that received a single 2000 mg/kg dose of *M. fragrans* extract, *A. lancea* extract, Prabchompoothaweep remedy extract, and those of the 7% Tween 80 group (negative control group) did not show statistically significant differences compared to the control group (untreated group) (*p* > 0.05) at the end of this study. Furthermore, the level of biochemical parameters of kidney functions, such as creatinine and BUN, in mice treated with 2000 mg/kg body weight of crude extracts and 7% Tween 80 showed no significant difference from those in mice in the control group (*p* > 0.05) (Table 9).

#### 3.5.3. Histological Examination of Liver and Kidney Tissues

**Figure 4:**
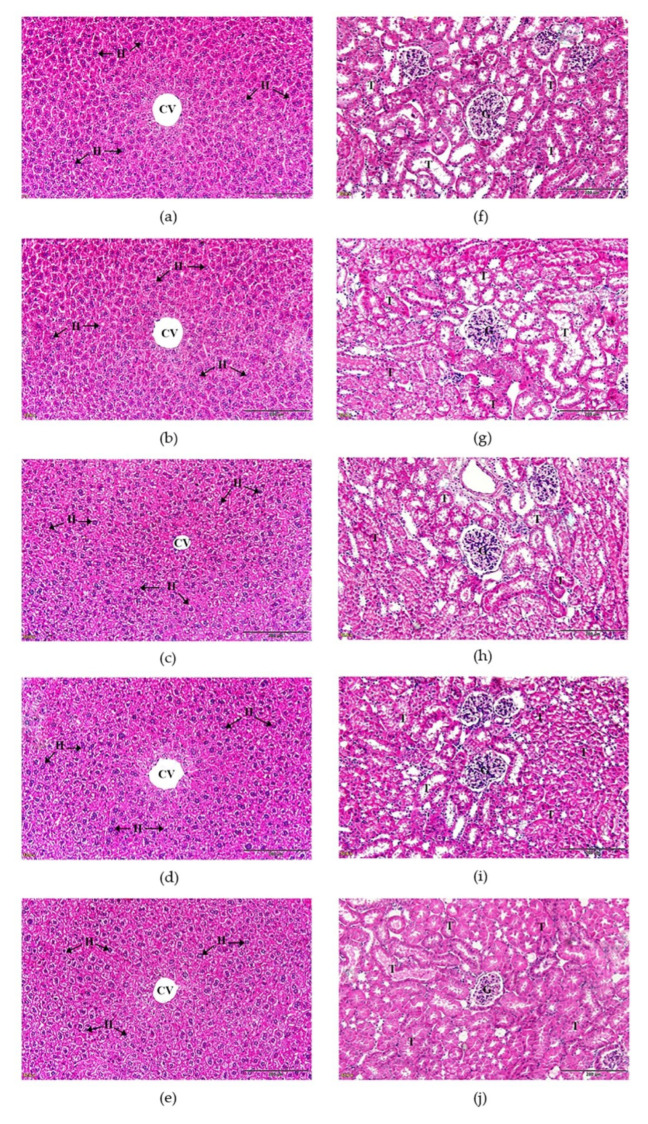
Histopathological examination of liver and kidney tissues from ICR mice that administrated with ethanolic extract from *M. fragrans*, *A. lancea*, and Prabchompoothaweep remedy in acute toxicity test: (**a**) histology of the liver tissue of control group, (**b**) histology of the liver tissue of 7% Tween 80 group, (**c**) histology of the liver tissue of *M. fragrans* treated mice, (**d**) histology of the liver tissue of *A. lancea* treated mice, (**e**) histology of the liver tissue of Prabchompoothaweep remedy treated mice, (**f**) histology of the kidney tissue of control group, (**g**) histology of the kidney tissue of 7% Tween 80 group, (**h**) histology of the kidney tissue of *M. fragrans* treated mice, (**i**) histology of the kidney tissue of *A. lancea* treated mice, (**j**) histology of the kidney tissue of Prabchompoothaweep remedy treated mice. All images were acquired at 20× magnification. Bar = 200 µm. CV, central vein; H, hepatocyte; T, tubule; G, glomerulus.

Histopathological examination of the liver and kidney samples is shown in Figure 4. The liver tissue morphology of the mice that received a single 2000 mg/kg dose of *M. fragrans* extract, *A. lancea* extract, and Prabchompoothaweep remedy extract manifested normal hepatocytes containing a red–pink cytoplasm and normal structures in the hepatic sinusoids and central vein. The sinusoidal vasodilation or inflammatory infiltration was not observed in the H&E staining of the liver tissue. Furthermore, the kidney morphology of the mice treated with a single dose of the crude extract revealed a normal structure of the glomerulus, Bowman’s capsule, and kidney epithelial cells compared to those of the control group (Figure 4f) and the 7% Tween 80 group (Figure 4g).

## 4. Discussion

Antimalarial treatment remains a public health concern in several countries. The use of traditional medicine that is safe, effective, and cost-efficient is a way to ensure that all patients have access to treatment [8]. From 2014 to 2023, the World Health Organization’s traditional medicine strategy has become popular worldwide and constantly increased each year [27]. Furthermore, natural plants are important sources of bioactive compounds, and many studies have focused on finding new substances to solve the antimalarial drug problem [24]. Therefore, this study focused on natural plants to stimulate the development of a new, effective antimalarial agent. In our previous report, in vitro studies showed that the ethanolic extracts of the mace of *M. fragrans*, rhizome of *A. lancea,* and Prabchompoothaweep remedy had anti-plasmodium activity against the *P. falciparum* K1 strain, with IC_50_ values of 5.96, 7.37, and 14.13 µg/mL, respectively (unpublished data). All IC_50_ values of the crude extracts were categorized as a good or promising activity for antimalarial effects [28]. A selectivity index (SI), which is calculated from the ratio between the toxic concentration to human cells (CC_50_) and the effective concentration to prevent parasite growth (IC_50_), which is lower than two, indicates the general toxicity of the compound [29]. These results showed that the ethanolic extract of the mace of *M. fragrans*, rhizome of *A. lancea*, and Prabchompoothaweep remedy exhibited SI values higher than two. Because the ethanolic extracts of *M. fragrans*, *A. lancea*, and Prabchompoothaweep remedy showed strong in vitro therapeutic effects with promising antimalarial activity and low toxicity to human cells, these two plants and one remedy were considered for in vivo antimalarial evaluation in this study. An in vivo model is commonly used to investigate the effects of a prodrug, the elimination of parasites by the immune system and the safety of the drug before processing into the clinical phase [30]. Mouse models have been used to identify a large number of conventional antimalarial agents, including chloroquine, halofantrine, mefloquine, and artemisinin derivatives [31]. In this study, ICR mice were inoculated with the wild-type *P. berghei* ANKA strain, a common model for the induction of malaria in mice and evaluation of antimalarial effects. The *P. berghei* ANKA strain is a suitable parasite that has higher accessibility and can sequester within the blood microcirculation. In this study, we used the 4-day suppressive test because it is a commonly used method for testing the antimalarial effects of candidate compounds in early infection. Moreover, this model shows the most reliable parameters, such as percentage of suppression of blood parasitemia [32].

In the present study, the 4-day suppressive test showed inhibition of parasitemia, which showed a high percentage in mice receiving 600 mg/kg *M. fragrans* (46.86%), 600 mg/kg Prabchompoothaweep remedy (60.11%), while *A. lancea* showed a high percentage of suppression in mice receiving 400 mg/kg (60.09%). *A. lancea* showed a high percentage of suppression in mice at a dose of 400 mg/kg because of its immunomodulatory property. Normally, cytokines play a major role in modulating the symptoms of malaria, parasitemia load, and the severity of malaria disease [33]. Moreover, the pro-inflammatory cytokines such as TNF-α, and IL-6 have been associated with severe malaria and death [34]. A previous study found that the low concentration of atractylodin, which is a bioactive compound of *A. Lancea*, significantly inhibited the expression of both TNF and IL-6, while the high concentration of atractylodin significantly suppressed only IL-6 expression [35]. Consistent with our results, the crude extract was identified as a considered active when parasitemia suppression was more than 30% [31]. Therefore, it can be implied that these crude extracts are active in schizonticide activity against *P. berghei* ANKA-infected mice. The antimalarial effects of the crude extract are associated with bioactive compounds such as polyphenols, flavonoids, alkaloids, terpenoids, and saponins [36]. Therefore, the antimalarial effect of ethanolic crude extracts could be due to a single or combined mechanism of action of these active compounds [37]. 

The results of phytochemical screening revealed that the ethanolic extract of *M. fragrans*, *A. lancea*, and Prabchompoothaweep remedy is rich in several plant secondary metabolites. *M. fragrans* extract contained flavonoids, terpenoids, alkaloids, and coumarins, while the extract of *A. lancea* contained terpenoids, alkaloids, and coumarins, and Prabchompoothaweep remedy contained terpenoid, alkaloids, tannins, and coumarins, all of which are associated with antimalarial activity. These results were consistent with a previous study on secondary plant metabolites. They have shown antimalarial activities posed by the classes of alkaloids, terpenes, flavonoids, xanthones, anthraquinones, phenolic compounds, sesquiterpenes, and other compounds [38,39]. The phytochemical constituents of the ethanolic extracts of *M. fragrans*, *A. lancea*, and Prabchompoothaweep remedy may have a single or synergistic effect to provide antimalarial properties through various mechanisms. In this context, flavonoids have been shown to prevent the transportation of L-glutamine and myoinositol into infected red blood cells, which play a role in parasite growth [40], while terpenoids (e.g., artemisinin) may exert their effect by the endoperoxidation that forms potentially toxic heme-adducts. Alkaloids (e.g., quinine) act as antimalarial agents by inhibiting protein synthesis and preventing heme (toxic) from being converted into hemozoin pigments (non-toxic) in parasite food vacuole [41]. Consequently, tannins also exhibit antimalarial effects by scavenging free radicals. Furthermore, coumarin compounds might contribute to antiplasmodial activity by controlling oxidative enzymes, such as superoxide dismutase, and inhibiting DNA synthesis. The antioxidant effects can disrupt heme polymerization, which oxidizes heme before heme polymerization, and unpolymerized heme is toxic to intraerythrocytic parasites [10]. Furthermore, phytochemical constituents, such as steroids, flavonoids, and other components, might act as antimalarial agents not only by directly attacking parasites but also by indirectly modulating the immune system of the host [42]. Therefore, the antiplasmodial activity observed in plants could have been derived from a single or synergistic effect of these metabolites.

Qualitative analysis of *M. fragrans* mace extracts presented many compounds. *M. fragrans* is an important source of secondary compounds consisting of coumarin (edulisin III), and flavonoids (kaempferol, (7′x,8′x)-4,7′-epoxy-3,8′-bilign-7-ene-3,5′-dimethoxy-4′,9,9′-triol), including citric acid and propyl 2-furanacrylate (fatty acid esters). Analysis of *A. lancea* rhizome extracts revealed the presence of polyphenols (chlorogenic acid), hydroxyanthraquinones (1,2,6,8-Tetrahydroxy-3-methylanthraquinone 2-*O*-b-D-glucoside), sesquiterpene lactone (taraxacolide 1-O-b-D-glucopyranoside), and salicylic acid. Furthermore, the Prabchompoothaweep remedy extracts allowed us to putatively identify 10 major peaks. The results showed the presence of flavonoid (luteolin), coumarins (6′-O-formylmarmin), and phenolic compounds (caffeic acid, eudesmic acid, gallic acid, and ellagic acid) constituents in the remedy. Some constituents analyzed by LC-MS are biologically active compounds. Luteolin has been shown to possess anti-inflammatory, antiallergy, anticancer, and antioxidant activity [43]. In addition, vanillic acid has been shown to exhibit anti-inflammatory and antioxidant effects both in vitro and in vivo in a carrageenan-induced inflammation model, and it has also shown anticancer, antifungal, antibacterial, and anti-viral effects [44,45]. Kaempferol has several pharmacological effects, including antioxidant and antibacterial activities [46]. Caffeic acid has potential as an antioxidant, anti-inflammatory, and antineoplastic agent [47]. Gallic acid exhibits antibacterial, anticancer, and antiplasmodial activities [48]. Among the identified compounds, ellagic acid has been reported to have anti-plasmodium properties. A previous study by Verotta et al. found that ellagic acid isolated from *Tristaniopsis callobuxus* (*Myrtaceae*) showed significant antiplasmodial activity against the resistant strain of *Plasmodium*, with an IC_50_ between 0.331 and 0.480 µM [49]. A study by Banzouzi et al. suggested that ellagic acid has also shown anti-plasmodium in mice infected with *Plasmodium vinckei pettri* using the Peter’s test [50]. Furthermore, Soh et al. found that ellagic acid inhibits parasitemia in a dose-dependent manner, with 50% suppression in mice receiving 1 mg/kg and 100% suppression in mice receiving 50 and 100 mg/kg via the intraperitoneal route [51]. In addition to antimalarial activity, ellagic acid also shows antioxidant properties and anti-inflammation that could prolong the survival rate after the administration of *T. albida* in experimental cerebral malaria (ECM) [52]. Flavonoid compounds also have antimalarial effects by stimulating the immune system, inhibiting the synthesis of fatty acids in parasites and preventing protein synthesis [53]. Therefore, the selected crude extract of the Prabchompoothaweep remedy might be responsible, at least partially, for antimalarial property, which is produced by a single phytoconstituent or the synergistic effect of these compounds, as mentioned above. However, further studies are needed to isolate, identify, and characterize active compounds, as well as to understand the mechanism of inhibition.

A decrease in PCV is one of the characteristics of malaria infection in mice. PCV was determined to investigate the effectiveness of the ethanolic crude extract in inhibiting erythrocyte damage caused by an increase in parasitemia [54]. To prevent PCV reduction, plants with antimalarial activity are expected to maintain PCV during mouse infection. Surprisingly, in a 4-day suppressive test, the ethanolic extract of *M. fragrans*, *A. lancea*, and Prabchompoothaweep remedy at all doses prevented PCV loss compared to the negative control group. It is possible that phenols and other metabolites in plants have antioxidant effects and membrane protection. Phenolic compounds have excellent antioxidant effects due to their hydroxyl groups, which can donate electrons to reactive oxygen species (ROS) [55]. The protective effect of the crude extracts was consistent with the results of studies by Wannang et al. [56], Saba et al. [57], and Misganaw et al. [58]. Moreover, the prevention of PCV reduction may be due to the absence of saponins in this crude extract. Normally, saponins act as phytodetergents, leading to cholesterol release from the cell membrane and promoting the permeability of the red blood cell membrane with strong hemolytic activity [59]. The effect of the plant extract on PCV loss may be due to the elimination of parasites from infected erythrocytes before hemolysis. Furthermore, the activation of the immune system and release of free radicals and ROS caused by malaria infection contribute to the degradation of hemoglobin and development of anemia [60]. In addition, the antioxidant activity of crude extracts, especially polyphenolic compounds, may protect red blood cells (RBCs) from ROS and promote the survival rate of both normal and infected RBCs during malaria infection.

The toxicity of the plant extracts was assessed using an oral acute toxicity test. Under these conditions, the mice received a single dose of 2000 mg/kg of the ethanolic extract of *M. fragrans*, *A. lancea*, and Prabchompoothaweep remedy, where a single high dose is suggested for acute toxicity testing [23]. The results of acute toxicity of all crude extracts revealed that there was no mortality and no signs of toxicity over 14 days. Therefore, the approximate median lethal dose (LD_50_) of the crude extracts was greater than 2000 mg/kg. According to the OECD’s Globally Harmonized System of Classification, crude extracts presented a low acute toxicity hazard with a category 5 classification. The results observed in the acute toxicity study with *M. fragrans* and *A. lancea* remedies are consistent with those of a previous study, indicating the safety profiles of this crude extract in a broad range of dose levels (1000−5000 mg/kg body weight) [17,61]. Food and water uptake were recorded to monitor toxicity because these parameters can be used to identify the harmful effects of crude extracts [62]. These results indicated that food and water consumption were not significantly different between the treatment and control groups. Body weight loss is a sensitive toxicity index after exposure to toxic compounds. In the acute toxicity test study, all treatments with *M. fragrans*, *A. lancea*, and Prabchompoothaweep remedy did not show significant differences (*p* < 0.05) in body weight loss compared with the control group on day 14. This observation suggests that the crude extracts did not disturb metabolism in these animals. In addition, the functions of the liver and kidneys were examined using biochemical analyses. Liver abnormalities were indicated by AST, ALT, and ALP levels. Damage to liver cells depends on the levels of AST, ALT, and ALP [63,64]. In all liver marker enzyme activities assessed, AST, ALT, and ALP levels were not significantly different between the treatment groups and the untreated control group. The levels of BUN and creatinine were analyzed for kidney function [65]. These results show that the levels of BUN and creatinine in mice receiving ethanolic extracts of *M. fragrans*, *A. lancea*, and Prabchompoothaweep remedy have normal functions in kidney organs that were not different from those in the untreated control group. Additionally, histopathological evaluation of kidney and liver tissue after treatment with the ethanolic extract of *M. fragrans*, *A. lancea*, and Prabchompoothaweep remedy did not show any abnormalities. Therefore, the results suggest that oral administration of crude extracts is neither harmful nor unsafe.

## 5. Conclusions

This study is the first to report the antimalarial activity of ethanolic extracts of *M. fragrans* mace and *A. lancea* rhizomes in a mouse model. The ethanolic crude extracts contained several phytoconstituents with important medicinal properties and antimalarial activity. The extracts significantly suppressed parasitemia. Moreover, the crude extracts also showed no adverse health effects on behavioral changes or liver or kidney function in the acute toxicity test. The overall results of this study illustrated that the use of rhizome extracts of *A. lancea* at 400 mg/kg body weight and extract of Prabchompoothaweep remedy at 600 mg/kg body weight could be developed as a new antimalarial drug treatment. More studies are required to isolate and identify the active compounds and to understand their mechanism of action.

## Figures and Tables

**Table 1:** Phytochemical screening of ethanolic extract of *M. fragrans* mace, *A. lancea* rhizome, and Prabchompoothaweep remedy.

Phytochemical Constituents	*M. fragrans*	*A. lancea*	Prabchompoothaweep Remedy
Flavonoid	+	-	-
Terpenoids	+	+	+
Alkaloids	+	+	+
Tannins	-	-	+
Anthraquinones	-	-	-
Cardiac glycosides	-	-	-
Saponins	-	-	-
Coumarins	+	-	+

(+), detected; (-), not detected phytochemical constituents.

**Table 2:** Compounds identified in the ethanolic *M. fragrans* extract by LC-QTOF-MS.

No.	M/Z	RT (min)	Compounds	Molecular Formula	Molecular Weight
1	133.014	2.087	Malic acid	C_4_H_6_O_5_	134.021
2	149.009	1.886	Tartaric acid	C_4_H_6_O_6_	150.016
3	285.040	27.493	Luteolin	C_15_H_10_O_6_	286.047
4	357.134	27.969	(7′x,8′x)-4,7′-Epoxy-3,8′-bilign-7-ene-3,5′-dimethoxy-4′,9,9′-triol	C_20_H_22_O_6_	358.141
5	201.149	31.852	3-Hydroxynonyl acetate	C_11_H_22_O_3_	202.156
6	265.056	2.1360	Monoglyceride citrate	C_9_H_14_O_9_	266.063
7	373.165	38.781	Sonchifolin	C_21_H_26_O_6_	374.172
8	345.134	31.690	Gibberellin A92	C_19_H_22_O_6_	346.141
9	161.045	4.429	3-Hydroxy-3-methyl-glutaric acid	C_6_H_10_O_5_	162.052
10	179.071	15.867	Propyl 2-furanacrylate	C_10_H_12_O_3_	180.078
11	207.066	18.673	Sinapyl aldehyde	C_11_H_12_O_4_	208.073
12	219.050	5.294	1-Hydroxypentane-1,2,5-tricarboxylate	C_8_H_12_O_7_	220.058
13	329.103	35.937	Isoamericanol A	C_18_H_18_O_6_	330.110
14	299.092	33.581	2,4-Dihydroxy-6,4′-dimethoxychalcone	C_17_H_16_O_5_	300.099
15	167.034	9.353	Dihydroxyphenylacetic acid	C_8_H_8_O_4_	168.042
16	183.102	34.258	Ascariadole epoxide	C_10_H_16_O_3_	184.109
17	375.144	31.978	alpha-Peroxyachifolide	C_20_H_24_O_7_	376.152
18	191.019	2.124	Citric acid	C_6_H_8_O_7_	192.026
19	287.055	21.191	3′,4′,5,7-Tetrahydroxyisoflavanone	C_15_H_12_O_6_	288.063
20	149.060	26.290	2-(2-Furanyl)-3-methyl-2-butenal	C_9_H_10_O_2_	150.067
21	329.139	36.576	Tetrahydrosappanone A trimethyl ether	C_19_H_22_O_5_	330.146
22	371.186	34.358	Tanabalin	C_22_H_28_O_5_	372.193
23	315.123	39.745	5′-Hydroxy-3′,4′,7-trimethoxyflavan	C_18_H_20_O_5_	316.130
24	265.144	38.918	Isoleptospermone	C_15_H_22_O_4_	266.151
25	237.113	20.077	Benzyl b-L-arabinopyranoside	C_13_H_18_O_4_	238.120
26	301.035	27.794	Hieracin	C_15_H_10_O_7_	302.042
27	285.040	32.491	Kaempferol	C_15_H_10_O_6_	286.047
28	389.160	39.444	Rosmic acid	C_21_H_26_O_7_	390.167
29	271.060	29.798	Methylnorlichexanthone	C_15_H_12_O_5_	272.068
30	267.071	1.936	2(α-D-Mannosyl)-D-glycerate	C_9_H_16_O_9_	268.078
31	177.040	3.139	L-Sorbosone	C_6_H_10_O_6_	178.047
32	303.050	16.481	(±)-Taxifolin	C_15_H_12_O_7_	304.058
33	177.019	9.265	Esculetin	C_9_H_6_O_4_	178.026
34	359.149	35.899	6′-O-Formylmarmin	C_20_H_24_O_6_	360.156
35	387.144	31.364	Edulisin III	C_21_H_24_O_7_	388.151
36	331.118	23.709	5′,8-Dihydroxy-3′,4′,7-trimethoxyflavan	C_18_H_20_O_6_	332.125
37	359.076	34.408	Jaceidin	C_18_H_16_O_8_	360.083
38	271.060	31.401	(±)-Naringenin	C_15_H_12_O_5_	272.067
39	201.112	6.847	2,6-Dimethyl-1,8-octanedioic acid	C_10_H_18_O_4_	202.120
40	329.232	33.243	9S,10S,11R-trihydroxy-12Z-octadecenoic acid	C_18_H_34_O_5_	330.240
41	311.128	39.657	Gancaonin V	C_19_H_20_O_4_	312.135
42	117.018	2.688	Succinic acid	C_4_H_6_O_4_	118.026
43	163.039	16.068	m-Coumaric acid	C_9_H_8_O_3_	164.047
44	197.045	9.904	2-Hydroxy-3,4-dimethoxybenzoic acid	C_9_H_10_O_5_	198.052
45	133.050	2.713	2,3-Dihydroxy-2-methylbutanoic acid	C_5_H_10_O_4_	134.057
46	281.138	10.129	Bisbynin	C_15_H_22_O_5_	282.146
47	221.081	13.762	2,3-Dihydro-3-hydroxy-6-methoxy-2,2-dimethyl-4H-1-benzopyran-4-one	C_12_H_14_O_4_	222.088
48	239.070	37.691	2,4-Dihydroxychalcone	C_15_H_12_O_3_	240.078
49	317.066	21.617	Dihydroisorhamnetin	C_16_H_14_O_7_	318.073
50	443.191	5.794	Cynaroside A	C_21_H_32_O_10_	444.198
51	371.134	20.577	Citrusin E	C_17_H_24_O_9_	372.141
52	447.092	18.473	Kaempferol-7-O-glucoside	C_21_H_20_O_11_	448.099
53	205.086	32.679	2,3-Dihydro-6-methoxy-2,2-dimethyl-4H-1-benzopyran-4-one	C_12_H_14_O_3_	206.094
54	343.154	31.101	Safficinolide	C_20_H_24_O_5_	344.161
55	353.102	32.303	1-(3,4-Dihydroxyphenyl)-7-(4-hydroxy-3-methoxyphenyl)-1,6-heptadiene-3,5-dione	C_20_H_18_O_6_	354.109
56	313.107	33.706	7-Hydroxyenterolactone	C_18_H_18_O_5_	314.114
57	445.170	11.357	Crosatoside B	C_20_H_30_O_11_	446.177
58	331.115	24.787	Mytilin A	C_13_H_20_N_2_O_8_	332.122
59	263.128	24.737	(+)-Abscisic acid	C_15_H_20_O_4_	264.135
60	426.227	35.285	Dihydroxyacidissiminol	C_25_H_33_NO_5_	427.234
61	343.118	22.682	Diosbulbin B	C_19_H_20_O_6_	344.125
62	187.096	19.475	Methyl N-(a-methylbutyryl) glycine	C_9_H_16_O_4_	188.104

**Table 3:** Compounds identified in the ethanolic *A. lancea* extract by LC-QTOF-MS.

No.	M/Z	RT (min)	Compounds	Molecular Formula	Molecular Weight
1	191.056	1.970	Quinic acid	C_7_H_12_O_6_	192.063
2	179.035	9.637	Caffeic acid	C_9_H_8_O_4_	180.042
3	177.019	9.274	Esculetin	C_9_H_6_O_4_	178.026
4	243.062	1.995	Pseudouridine	C_9_H_12_N_2_O_6_	244.069
5	191.034	14.798	Scopoletin	C_10_H_8_O_4_	192.042
6	209.118	33.628	3-Ethenyl-2,5-dimethyl-4-oxohex-5-en-2-yl acetate	C_12_H_18_O_3_	210.125
7	161.024	14.673	3-Hydroxycoumarin	C_9_H_6_O_3_	162.031
8	353.087	7.419	Chlorogenic acid	C_16_H_18_O_9_	354.094
9	281.139	32.475	Bisbynin	C_15_H_22_O_5_	282.146
10	207.029	11.341	Fraxetin	C_10_H_8_O_5_	208.036
11	207.066	28.153	5-(3′,5′-Dihydroxyphenyl)-gamma-valerolactone	C_11_H_12_O_4_	208.073
12	265.144	33.653	Isoleptospermone	C_15_H_22_O_4_	266.151
13	193.050	15.124	Scytalone	C_10_H_10_O_4_	194.057
14	311.128	28.454	Gancaonin V	C_19_H_20_O_4_	312.135
15	153.019	8.372	Gentisic acid	C_7_H_6_O_4_	154.026
16	147.029	2.721	D-threo-3-methylmalate	C_5_H_8_O_5_	148.036
17	341.108	1.907	Sucrose	C_12_H_22_O_11_	342.115
18	353.087	8.008	5Z-Caffeoylquinic acid	C_16_H_18_O_9_	354.094
19	341.087	7.156	Glucocaffeic acid	C_15_H_18_O_9_	342.094
20	447.092	12.368	1,2,6,8-Tetrahydroxy-3-methylanthraquinone 2-*O*-b-D-glucoside	C_21_H_20_O_11_	448.100
21	427.196	16.703	Taraxacolide 1-O-b-D-glucopyranoside	C_21_H_32_O_9_	428.204
22	225.112	25.472	3,7-Dimethyl-2E,6E-decadien-1,10-dioic acid	C_12_H_18_O_4_	226.120
23	221.045	15.487	Isofraxidin	C_11_H_10_O_5_	222.052
24	128.035	2.208	Pyroglutamic acid	C_5_H_7_NO_3_	129.042
25	381.175	10.163	1,2,10-Trihydroxydihydro-trans-linalyl oxide 7-O-beta-D-glucopyranoside	C_16_H_30_O_10_	382.183
26	337.092	10.338	Hydrojuglone glucoside	C_16_H_18_O_8_	338.099
27	441.175	15.600	Lusitanicoside	C_21_H_30_O_10_	442.183
28	485.199	14.673	Glucosylgalactosyl hydroxylysine	C_18_H_34_N_2_O_13_	486.207
29	401.144	8.847	Benzyl O-(arabinofuranosyl-(1->6)-glucoside)	C_18_H_26_O_10_	402.151
30	385.164	26.675	Gingerenone B	C_22_H_26_O_6_	386.172
31	335.076	13.169	4-O-Caffeoylshikimic acid	C_16_H_16_O_8_	336.083
32	305.138	32.788	Achillicin	C_17_H_22_O_5_	306.146
33	425.144	16.414	6-(2-Carboxyethyl)-7-hydroxy-2,2-dimethyl-4-chromanone glucoside	C_20_H_26_O_10_	426.151
34	353.144	26.675	Isopropyl apiosylglucoside	C_14_H_26_O_10_	354.151
35	503.175	13.169	(S)-Multifidol 2-(apiosyl-(1->6)-glucoside)	C_22_H_32_O_13_	504.183
36	329.232	33.164	9S,10S,11R-trihydroxy-12Z-octadecenoic acid	C_18_H_34_O_5_	330.239
37	461.238	13.270	xi-Linalool 3-(rhamnosyl-(1->6)-glucoside)	C_22_H_38_O_10_	462.245
38	511.238	10.789	3-O-(beta-D-glucopyranosyl-(1->6)-beta-D-glucopyranosyl) ethyl 3-hydroxyoctanoate	C_22_H_40_O_13_	512.245
39	393.133	39.996	Rotenone	C_23_H_22_O_6_	394.141
40	447.092	15.976	Kaempferol-7-O-glucoside	C_21_H_20_O_11_	448.099
41	461.165	9.837	Verbasoside	C_20_H_30_O_12_	462.172
42	479.248	10.263	3-O-(alpha-L-rhamnopyranosyl-(1-2)-alpha-L-rhamnopyranosyl)-3-hydroxydecanoic acid	C_22_H_40_O_11_	480.255
43	529.264	18.356	Cinncassiol D2 glucoside	C_26_H_42_O_11_	530.271
44	515.118	20.235	3″,4″-Diacetylafzelin	C_25_H_24_O_12_	516.125
45	128.035	2.496	(r)-(+)-2-Pyrrolidone-5-carboxylic acid	C_5_H_7_NO_3_	129.042
46	441.212	16.828	CAY10509	C_23_H_35_FO_5_S	442.219
47	299.141	32.688	Bifenazate	C_17_H_20_N_2_O_3_	300.148

**Table 4:** Compounds identified in the ethanolic extract of Prabchompoothaweep remedy by LC-QTOF-MS.

No.	M/Z	RT (min)	Compounds	Molecular Formula	Molecular Weight
1	173.045	1.988	Shikimic acid	C_7_H_10_O_5_	174.052
2	197.045	9.842	2-Hydroxy-3,4-dimethoxybenzoic acid	C_9_H_10_O_5_	198.052
3	169.014	3.816	Gallic acid	C_7_H_6_O_5_	170.021
4	177.019	9.216	Esculetin	C_9_H_6_O_4_	178.026
5	137.024	7.725	3,4-Dihydroxybenzaldehyde	C_7_H_6_O_3_	138.031
6	169.014	6.497	2,4,6-Trihydroxybenzoic acid	C_7_H_6_O_5_	170.021
7	243.051	3.491	1-O-Galloylglycerol	C_10_H_12_O_7_	244.058
8	166.050	7.976	2-Amino-3-methoxy-benzoic acid	C_8_H_9_NO_3_	167.058
9	447.129	24.625	Piperenol C	C_22_H_24_O_10_	448.136
10	153.019	5.320	3,4-Dihydroxybenzoic acid	C_7_H_6_O_4_	154.026
11	211.061	19.401	Eudesmic acid	C_10_H_12_O_5_	212.068
12	187.097	19.489	Methyl N-(a-methylbutyryl) glycine	C_9_H_16_O_4_	188.104
13	197.045	13.212	3,4-O-Dimethylgallic acid	C_9_H_10_O_5_	198.052
14	313.056	3.290	Salicyl phenolic glucuronide	C_13_H_14_O_9_	314.063
15	161.081	7.249	Potassium 2-(1’-ethoxy) ethoxypropanoate	C_7_H_14_O_4_	162.089
16	179.035	9.629	Caffeic acid	C_9_H_8_O_4_	180.042
17	151.040	9.078	4-Acetoxyphenol	C_8_H_8_O_3_	152.047
18	290.088	2.012	Sarmentosin epoxide	C_11_H_17_NO_8_	291.095
19	191.034	22.608	5,7-Dihydroxy-4-methylcoumarin	C_10_H_8_O_4_	192.042
20	353.087	7.713	5Z-Caffeoylquinic acid	C_16_H_18_O_9_	354.094
21	237.113	20.065	Benzyl b-L-arabinopyranoside	C_13_H_18_O_4_	238.120
22	222.040	14.340	(R)-2,3-Dihydro-3,5-dihydroxy-2-oxo-3-indoleacetic acid	C_10_H_9_NO_5_	223.047
23	218.103	3.516	Pantothenic acid	C_9_H_17_NO_5_	219.110
24	195.102	16.169	Isobutyl 2-furanpropionate	C_11_H_16_O_3_	196.109
25	421.186	32.869	Picrasin F	C_22_H_30_O_8_	422.193
26	355.030	2.137	(+)-Chebulic acid	C_14_H_12_O_11_	356.037
27	153.019	8.352	Gentisic acid	C_7_H_6_O_4_	154.026
28	325.056	3.992	Fertaric acid	C_14_H_14_O_9_	326.063
29	243.123	23.360	Polyethylene, oxidized	C_12_H_20_O_5_	244.130
30	233.045	6.735	7-Hydroxy-2-methyl-4-oxo-4H-1-benzopyran-5-acetic acid	C_12_H_10_O_5_	234.052
31	299.055	32.242	Diosmetin	C_16_H_12_O_6_	300.063
32	310.140	11.734	Leonurine	C_14_H_21_N_3_O_5_	311.147
33	300.998	15.430	Ellagic acid	C_14_H_6_O_8_	302.005
34	328.118	20.604	N-trans-Feruloyloctopamine	C_18_H_19_NO_5_	329.125
35	225.112	11.947	3,7-Dimethyl-2E,6E-decadien-1,10-dioic acid	C_12_H_18_O_4_	226.120
36	163.039	13.538	m-Coumaric acid	C_9_H_8_O_3_	164.047
37	321.024	7.900	Digallate	C_14_H_10_O_9_	322.032
38	359.149	34.898	6′-O-Formylmarmin	C_20_H_24_O_6_	360.156
39	651.083	9.316	Amlaic acid	C_27_H_24_O_19_	652.090
40	285.040	32.518	Kaempferol	C_15_H_10_O_6_	286.047
41	463.087	15.881	Quercetin 3-galactoside	C_21_H_20_O_12_	464.094
42	347.076	7.024	alpha-(1,2-Dihydroxyethyl)-1,2,3,4-tetrahydro-7-hydroxy-9-methoxy-3,4-dioxocyclopenta(c) benzopyran-6-acetaldehyde	C_17_H_16_O_8_	348.083
43	431.170	33.708	Melledonal A	C_23_H_28_O_8_	432.177
44	261.040	17.396	2-Acetyl-5,8-dihydroxy-3-methoxy-1,4-naphthoquinone	C_13_H_10_O_6_	262.047
45	315.050	33.082	1,3,5,8-Tetrahydroxy-6-methoxy-2-methylanthraquinone	C_16_H_12_O_7_	316.057
46	326.087	7.449	Blepharin	C_14_H_17_NO_8_	327.094
47	461.108	25.966	Rhamnetin 3-rhamnoside	C_22_H_22_O_11_	462.115
48	161.060	18.223	Allyl benzoate	C_10_H_10_O_2_	162.067
49	128.035	2.426	Pyroglutamic acid	C_5_H_7_NO_3_	129.042
50	271.060	31.302	(±)-Naringenin	C_15_H_12_O_5_	272.067
51	264.066	34.710	Piperolactam A	C_16_H_11_NO_3_	265.073
52	272.129	29.724	(2E)-Piperamide-C5:1	C_16_H_19_NO_3_	273.136
53	191.055	1.887	Quinic acid	C_7_H_12_O_6_	192.063
54	134.024	10.356	2-Benzoxazolol	C_7_H_5_NO_2_	135.032
55	361.165	22.821	Gibberellin A98	C_20_H_26_O_6_	362.172
56	201.112	26.041	2,6-Dimethyl-1,8-octanedioic acid	C_10_H_18_O_4_	202.120
57	476.040	13.688	Isoterchebin	C_41_H_30_O_27_	954.096
58	312.123	24.863	Pterostilbene glycinate	C_18_H_19_NO_4_	313.131
59	285.040	27.444	Luteolin	C_15_H_10_O_6_	286.047
60	635.088	11.308	3-O-Galloylhamamelitannin	C_27_H_24_O_18_	636.095
61	351.053	26.191	4′-O-Methyl-(-)-epicatechin-7-O-sulfate	C_16_H_16_O_7_S	352.061
62	269.045	31.453	Apigenin	C_15_H_10_O_5_	270.052
63	343.045	36.564	Aflatoxin GM1	C_17_H_12_O_8_	344.052
64	307.081	18.474	4R,5R,6S-Trihydroxy-2-hydroxymethyl-2-cyclohexen-1-one 6-(2-hydroxy-6-methylbenzoate)	C_15_H_16_O_7_	308.089
65	447.092	18.487	Kaempferol-7-O-glucoside	C_21_H_20_O_11_	448.099
66	461.072	19.726	3-Methylellagic acid 8-rhamnoside	C_21_H_18_O_12_	462.079
67	201.018	26.341	6-Hydroxyangelicin	C_11_H_6_O_4_	202.026
68	623.197	15.943	Isoacteoside	C_29_H_36_O_15_	624.204
69	211.060	5.119	3-Hydroxy-4-methoxyphenyllactic acid	C_10_H_12_O_5_	212.067
70	301.034	27.820	Hieracin	C_15_H_10_O_7_	302.042
71	477.139	24.462	Eugenol O-[3,4,5-Trihydroxybenzoyl-(->6)-b-D-glucopyranoside]	C_23_H_26_O_11_	478.146
72	342.134	25.665	N-trans-Feruloyl-4-O-methyldopamine	C_19_H_21_NO_5_	343.141
73	547.144	21.255	Puerarin xyloside	C_26_H_28_O_13_	548.152
74	251.128	15.667	QH (2)	C_14_H_20_O_4_	252.135
75	329.029	28.897	2,8-Di-O-methylellagic acid	C_16_H_10_O_8_	330.036
76	256.133	34.309	Coumaperine	C_16_H_19_NO_2_	257.140
77	491.118	28.772	3′,7-Dimethoxy-4′,5,8-trihydroxyflavone 8-glucoside	C_23_H_24_O_12_	492.125
78	281.138	10.080	Bisbynin	C_15_H_22_O_5_	282.146
79	403.175	32.255	Myristicanol B	C_22_H_28_O_7_	404.183
80	403.123	5.921	Oleoside 11-methyl ester	C_17_H_24_O_11_	404.131
81	465.102	17.998	(-)-Epicatechin 7-O-glucuronide	C_21_H_22_O_12_	466.110
82	379.175	20.403	6b-Angeloyl-3b,8b,9b-trihydroxy-7(11)-eremophilen-12,8-olide	C_20_H_28_O_7_	380.182
83	593.150	17.221	Saponarin	C_27_H_30_O_15_	594.157
84	379.012	28.672	Tectorigenin 7-sulfate	C_16_H_12_O_9_S	380.019
85	241.071	6.046	Elenaic acid	C_11_H_14_O_6_	242.078
86	955.104	16.570	Chebulinic acid	C_41_H_32_O_27_	956.111
87	477.102	19.000	Myricetin 3,4′-dimethyl ether 3′-xyloside	C_22_H_22_O_12_	478.110
88	497.223	19.614	2-O-(beta-D-galactopyranosyl-(1->6)-beta-D-galactopyranosyl) 2S-hydroxynonanoic acid	C_21_H_38_O_13_	498.230
89	593.129	27.795	6″-O-p-Coumaroyltrifolin	C_30_H_26_O_13_	594.136
90	515.118	20.177	3″,4″-Diacetylafzelin	C_25_H_24_O_12_	516.125
91	161.045	4.443	3-Hydroxy-3-methyl-glutaric acid	C_6_H_10_O_5_	162.052
92	447.092	12.385	1,2,6,8-Tetrahydroxy-3-methylanthraquinone 2-*O*-b-D-glucoside	C_21_H_20_O_11_	448.099
93	387.107	16.945	7-Hydroxy-3′,4′,5,6,8-pentamethoxyflavone	C_20_H_20_O_8_	388.115
94	769.254	18.349	Leonoside A	C_35_H_46_O_19_	770.261
95	637.176	13.876	Quercetin 3,3′-dimethyl ether 7-rutinoside	C_29_H_34_O_16_	638.183
96	431.097	19.150	Apigenin 7-O-glucoside	C_21_H_20_O_10_	432.104
97	581.222	12.498	(+)-Lyoniresinol 9-glucoside	C_28_H_38_O_13_	582.230
98	461.238	13.976	xi-Linalool 3-(rhamnosyl-(1->6)-glucoside)	C_22_H_38_O_10_	462.245
99	695.399	31.177	Glucosyl passiflorate	C_37_H_60_O_12_	696.407
100	461.165	4.543	Verbasoside	C_20_H_30_O_12_	462.172
101	755.238	15.292	Hesperetin 7-(2,6-dirhamnosylglucoside)	C_34_H_44_O_19_	756.246
102	435.128	19.075	Phenethyl 6-galloylglucoside	C_21_H_24_O_10_	436.135
103	429.152	23.685	2,3-dinor Fluprostenol	C_21_H_25_F_3_O_6_	430.159
104	651.228	24.262	(-)-Matairesinol 4′-(apiosyl-(1->2)-glucoside)	C_31_H_40_O_15_	652.235
105	153.055	4.944	2-Furanylmethyl propanoate	C_8_H_10_O_3_	154.062
106	665.207	23.460	Tetramethylquercetin 3-rutinoside	C_31_H_38_O_16_	666.214
107	637.212	19.376	4′-Hydroxy-5,7,2′-trimethoxyflavanone 4′-rhamnosyl-(1->6)-glucoside	C_30_H_38_O_15_	638.219
108	582.259	30.238	N1, N5, N10-Tricoumaroyl spermidine	C_34_H_37_N_3_O_6_	583.266
109	433.149	20.854	Vestitone 7-glucoside	C_22_H_26_O_9_	434.156
110	453.248	33.457	Rhodojaponin IV	C_24_H_38_O_8_	454.255
111	137.024	8.088	m-Salicylic acid	C_7_H_6_O_3_	138.031
112	477.066	10.080	Quercetin 3′-O-glucuronide	C_21_H_18_O_13_	478.073
113	787.098	14.741	1,2’,3,5-Tetra-O-galloylhamamelofuranose	C_34_H_28_O_22_	788.105
114	577.154	17.647	Scutellarein 7,4′-dirhamnoside	C_27_H_30_O_14_	578.162
115	939.108	17.171	1,2,3,4,6-Pentakis-O-galloyl-beta-D-glucose	C_41_H_32_O_26_	940.115
116	331.081	16.795	2′,3,5-Trihydroxy-5′,7-dimethoxyflavanone	C_17_H_16_O_7_	332.088
117	347.037	3.941	2-(α-D-Mannosyl)-3-phosphoglycerate	C_9_H_17_O_12_P	348.045
118	315.159	34.910	Isopulegone caffeate	C_19_H_24_O_4_	316.166

**Table 5:** Effect of ethanolic extracts of *M. fragrans*, *A. lancea*, and Prabchompoothaweep remedy on parasite level and parasite suppression in the 4-day suppressive test.

Group	Dose (mg/kg)	% Parasitemia	% Suppression
7% Tween 80	-	40.45 ± 2.15 ^b,c,d,e,f,g,h,i,j,k,l^	-
Artesunate	6	2.18 ± 0.50 ^a,d,e,f,g,h,i,j,k,l^	95.32 ± 0.57 ^d,e,f,g,h,i,j,k,l^
Chloroquine	25	0.27 ± 0.15 ^a,d,e,f,g,h,i,j,k,l^	99.34 ± 0.37 ^d,e,f,g,h,i,j,k,l^
*M. fragrans*	200	24.94 ± 2.50 ^a,b,c,h,l^	38.32 ± 6.18 ^b,c,h,i,k,l^
	400	22.36 ± 1.26 ^a,b,c,h,l^	44.17 ± 3.12 ^b,c,h,l^
	600	21.48 ± 0.73 ^a,b,c,h,l^	46.86 ± 1.80 ^b,c,h,l^
*A. lancea*	200	21.53 ± 2.47 ^a,b,c,h,l^	46.75 ± 6.11 ^b,c,h,j,k,l^
	400	16.13 ± 0.41 ^a,b,c,d,e,f,g,i,j,k^	60.09 ± 1.03 ^b,c,d,e,f,g,i,j,k^
	600	20.91 ± 1.15 ^a,b,c,h,l^	48.29 ± 2.86 ^b,c,d,h,l^
Prabchompoothaweep remedy	200	24.60 ± 1.03 ^a,b,c,h,l^	39.18 ± 2.56 ^b,c,h,l^
	400	20.88 ± 3.08 ^a,b,c,h,l^	48.35 ± 7.62 ^b,c,d,h,l^
	600	16.13 ± 0.58 ^a,b,c,d,e,f,g,i,j,k^	60.11 ± 1.44 ^b,c,d,e,f,g,i,j,k^

Data are presented as mean ± SEM (n = 5 per group), *p* < 0.05. ^a^ Compared to the negative control, ^b^ compared to artesunate, ^c^ compared to chloroquine, ^d^ compared to 200 mg/kg of *M. fragrans*, ^e^ compared to 400 mg/kg of *M. fragrans*, ^f^ compared to 600 mg/kg of *M. fragrans*, ^g^ compared to 200 mg/kg of *A. lancea*, ^h^ compared to 400 mg/kg of *A. lancea*, ^i^ compared to 600 mg/kg of *A. lancea*, ^j^ compared to 200 mg/kg of Prabchompoothaweep remedy, ^k^ compared to 400 mg/kg of Prabchompoothaweep remedy, and ^l^ compared to 600 mg/kg of Prabchompoothaweep remedy.

**Table 6:** Effect of ethanolic extracts of *M. fragrans*, *A. lancea*, and Prabchompoothaweep remedy on pack cell volume in the 4-day suppressive test.

Group	Dose (mg/kg)	Day 0	Day 4	% Change
7% Tween 80	-	49.60 ± 1.01	45.00 ± 1.89	−10.35 ± 3.67% ^b,c^
Artesunate	6	52.20 ± 1.32	54.80 ± 1.16	4.74 ± 1.43% ^a,d,e,f,g,h,i,j,k,l^
Chloroquine	25	51.80 ± 0.43	53.40 ± 1.47	2.87 ± 2.36% ^a,d,j^
*M. fragrans*	200	54.00 ± 0.89	49.80 ± 2.03	−8.58 ± 3.89% ^b,c^
	400	51.20 ± 1.16	48.40 ± 0.80	−5.78 ± 1.52% ^b^
	600	51.00 ± 1.09	48.80 ± 1.93	−4.64 ± 4.05% ^b^
*A. lancea*	200	52.00 ± 2.73	49.40 ± 2.17	−5.58 ± 8.67% ^b^
	400	52.40 ± 1.01	50.00 ± 1.67	−4.86 ± 2.15% ^b^
	600	51.20 ± 1.16	48.80 ± 1.46	−5.01 ± 3.73% ^b^
Prabchompoothaweep remedy	200	52.60 ± 1.35	48.20 ± 2.13	−9.25 ± 3.46% ^b,c^
	400	51.80 ± 1.83	49.40 ± 1.35	−4.87 ± 3.02% ^b^
	600	50.80 ± 2.63	48.60 ± 1.62	−4.47 ± 2.65% ^b^

Data are presented as mean ± SEM (n = 5 per group), *p* < 0.05. ^a^ Compared to the negative control, ^b^ compared to artesunate, ^c^ compared to chloroquine, ^d^ compared to 200 mg/kg of *M. fragrans*, ^e^ compared to 400 mg/kg of *M. fragrans*, ^f^ compared to 600 mg/kg of *M. fragrans*, ^g^ compared to 200 mg/kg of *A. lancea*, ^h^ compared to 400 mg/kg of *A. lancea*, ^i^ compared to 600 mg/kg of *A. lancea*, ^j^ compared to 200 mg/kg of Prabchompoothaweep remedy, ^k^ compared to 400 mg/kg Prabchompoothaweep remedy, and ^l^ compared to 600 mg/kg of Prabchompoothaweep remedy.

**Table 7:** Effect of ethanolic extracts of *M. fragrans*, *A. lancea*, and Prabchompoothaweep remedy on food and water uptake in the acute toxicity test at week 1 and week 2 after treatment.

**Food Consumption (g)**	**Week 1**	**Week 2**
Normal mice	25.0 ± 3.5	21.6 ± 2.7
7% Tween 80	22.1 ± 1.7	20.8 ± 2.5
*M. fragrans* 2000 mg/kg	20.8 ± 2.0	20.7 ± 0.8
*A. lancea* 2000 mg/kg	22.8 ± 2.5	22.4 ± 2.3
Prabchompoothaweep remedy 2000 mg/kg	23.6 ± 3.9	22.0 ± 1.9
**Water Consumption (mL)**	**Week 1**	**Week 2**
Normal mice	122.2 ± 4.7	125.8 ± 7.9
7% Tween 80	122.4 ± 8.3	126.7 ± 8.3
*M. fragrans* 2000 mg/kg	122.4 ± 3.5	127.7 ± 3.5
*A. lancea* 2000 mg/kg	126.0 ± 4.8	130.4 ± 5.0
Prabchompoothaweep remedy 2000 mg/kg	125.0 ± 2.6	130.5 ± 4.8

Data are presented as mean ± SEM (n = 5 per group).

**Table 8:** Effect of ethanolic extracts of *M. fragrans*, *A. lancea*, and Prabchompoothaweep remedy on body weight changes in the acute toxicity test on day 0 and day 14 after treatment.

Group	Mean Body Weight		
	**Day 0**	**Day 14**	**% Change**
Normal mice	33.4 ± 1.5	39.2 ± 2.2	14.6 ± 1.7%
7% Tween 80	32.5 ± 1.4	36.5 ± 1.5	11.1 ± 1.3%
*M. fragrans* 2000 mg/kg	32.8 ± 1.2	38.0 ± 2.4	13.4 ± 3.3%
*A. lancea* 2000 mg/kg	33.0 ± 1.6	38.0 ± 2.7	13.0 ± 2.6%
Prabchompoothaweep remedy 2000 mg/kg	32.1 ± 1.1	36.8 ± 1.4	12.6 ± 1.5%

Data are presented as mean ± SEM (n = 5 per group).

**Table 9:** Effect of ethanolic extracts of *M. fragrans*, *A. lancea*, and Prabchompoothaweep remedy on kidney and liver functions in the acute toxicity test.

Parameters	Normal Mice	7% Tween 80	*M. fragrans*	*A. lancea*	Prabchompoothaweep Remedy
**Liver Function Test**					
AST (U/L)	83.80 ± 7.13	83.00 ± 9.18	87.75 ± 12.57	94.60 ± 8.77	92.00 ± 5.17
ALT (U/L)	36.80 ± 8.08	38.80 ± 3.70	31.75 ± 6.96	34.60 ± 5.57	34.60 ± 7.05
ALP (U/L)	92.10 ± 11.35	91.04 ± 7.86	90.50 ± 8.96	88.40 ± 7.03	89.20 ± 12.79
**Kidney Function Test**					
BUN (mg/dL)	26.42 ± 3.86	31.04 ± 3.96	25.45 ± 2.30	25.56 ± 3.89	25.26 ± 2.12
Creatinine (mg/dL)	0.66 ± 0.04	0.69 ± 0.07	0.66 ± 0.03	0.65 ± 0.04	0.61 ± 0.06

Data are presented as mean ± SEM (n = 5 per group).

## Data Availability

Data associated with this study have been included in this published article. Additional files are available from the corresponding authors upon request.
